# Outcomes and risk management in type B aortic dissection patients with acute kidney injury: a concise review

**DOI:** 10.1080/0886022X.2021.1905664

**Published:** 2021-03-30

**Authors:** Xiaolan Chen, Ming Bai, Shiren Sun, Xiangmei Chen

**Affiliations:** a The Nephrology Department, Xijing Hospital, The Fourth Military Medical University, Xi’an, PR China; b Department of Nephrology, State Key Laboratory of Kidney Disease, Chinese People’s Liberation Army General Hospital and Military Medical Postgraduate College, Beijing, PR China

**Keywords:** Acute kidney injury, type B aortic dissection, continuous renal replacement therapy, thoracic endovascular aortic repair

## Abstract

**Purpose:**

Type B aortic dissection is a rare but life-threatening disease. Thoracic endovascular aortic repair (TEVAR) was widely used for Type B aortic dissection patients in the last decade due to the lower mortality and morbidity compared with open chest surgical repair (OCSR). AKI in type B aortic dissection is a well-recognized complication and indicates poor short-term and long-term outcome. The objective of this concise review was to identify the risk factors and the impact of AKI on type B aortic dissection patients.

**Methods and results:**

A literature search was performed using PubMed, Embase, MEDLINE, and Cochrane Library with the search terms ‘type B aortic dissection’ and ‘acute kidney injury’ (AKI), and all English-language literatures published in print or available online from inception through August 2020 were thoroughly reviewed. Studies that reported relative AKI risks and outcomes in type B aortic dissection patient were included. Major mechanisms of AKI in type B aortic dissection included renal hypoperfusion, inflammation response, and the use of contrast medium. Type B aortic dissection patients with AKI significantly had increased hospital stay duration, need of renal replacement therapy, and 30-d and 1-year mortality.

**Conclusions:**

AKI in type B aortic dissection is a well-recognized complication and associated with poor short-term and long-term outcome. Early identification of high-risk patients, early diagnosis of AKI, stabilization of the hemodynamic parameters, avoidance of nephrotoxic drugs, and optimization of the use of contrast agents are the major strategies for the reduction of AKI in type B aortic dissection patients.

## Introduction

Aortic dissection has an incidence of 4.7–6/100,000 and causes 3000 deaths per year in the United States [1]. It has been estimated approximately one-third of aortic dissection is categorized as type B anatomically [1,2]. Type B aortic dissection involves the descending aorta distal to the left subclavian artery and its management is relatively complex. The emergent intervention is usually employed for type B aortic dissection patients complicated by malperfusion syndrome, rapid aortic expansion, or rupture in order to prevent poor outcomes. Recently, a meta-analysis also suggests thoracic endovascular aortic repair (TEVAR) to be an effective alternative to open repair with a lower morbidity [3].

Acute kidney injury (AKI) is a common complication with an incidence of 13–36% in type B aortic dissection patients and is believed to be associated with adverse outcomes [1,4–7]. It was reported that mild renal function deterioration was related to increased mortality in type B aortic dissection patients [8]. Additionally, the occurrence of AKI increased the 10-year mortality of type B aortic dissection patients regardless of other risk factors, even in patients with recovered renal function on hospital discharge [9]. However, the incidence, characteristics, and risk factors of AKI in type B aortic dissection patients treated medically or surgically are not adequately described [9–11]. Most researches have concentrated mainly on the types of conditions requiring TEVAR, such as transections to penetrating ulcers and aneurysms, dissections [12,13]. However, type B aortic dissection patients have other pathologic states that may affect renal function in many aspects: 1) renal perfusion may be affected by the blood supply for the side branch of the aorta from a true or false lumen [4]; 2) the renal function may be impacted the true lumen or false lumen hemodynamic change and inflammatory response after aortic stent-graft deployment [13]; 3) due to a complicated true lumen and false lumen association, more contrast agent may be administered to ensure that the stent graft is implanted in the true lumen and at the right place; and 4) the renal perfusion may be reduced because of the blood pressure change during the operation, such as controlling decompression during stent deployment. However, the evidence directly related to AKI in type B aortic dissection is limited. Currently, specific prevention and treatment strategies for AKI in type B aortic dissection are limited. Therefore, identification of the patients with a high AKI risk, early diagnosis of AKI, and appropriate renal support therapy are the key steps to decrease the development of AKI and to improve patient outcomes.

Therefore, we performed a literature search using PubMed, Embase, MEDLINE, and Cochrane Library with the search terms ‘type B aortic dissection’ and ‘acute kidney injury’, and all English-language literatures published in print or available online from inception through August 2020 were carefully reviewed. In the review, we summarized the current evidence on the epidemiology, pathophysiology, risk factors, and management of AKI in type B aortic dissection patients.

## Epidemiology and pathogenesis

### Epidemiology

The reported AKI incidence widely ranges from 17% to 21%, the main reasons for that were the wide variability in AKI definition criteria and patient epidemiology characteristics that were, until recently, used in the literature. Several investigators used only changes in serum creatinine (SCr) concentration as a marker of immediate (24–48 h) renal dysfunction, which was reported as AKI [9,14], not including urine output. Additionally, some studies included patients with medical, surgical, or endovascular treatment [9,14,15], while some studies included patients only treated with endovascular repair [10,11]. Overall, AKI is reported to have a lower incidence after endovascular stent graft than after open repair or medical management for type B aortic dissection [9].

For studies using the RIFLE criteria to report AKI, the incidence was 30.8% [10]; if the AKIN criteria were used, the incidence was 36% [16]. Of note, KDIGO criteria for AKI staging demonstrated greater sensitivity to recognize AKI and to predict in-hospital mortality compared with the RIFLE or AKIN criteria [17] (Table 1). AKI had been reported to occur at a rate between 30% and 52% based on the KDIGO criteria in patients with or without TEVAR [9,11,18,19]. Most type B aortic dissection patients who develop AKI are usually classified as Stage 1 in these series. Unfortunately, for patients who have underwent cardiac surgery, these criteria are problematic because fluid resuscitation is common, and fluid loading during surgery is universal. Thus, the application of these criteria after cardiac surgery without correction for SCr and urine output changes owing to fluid balance leads to underestimation of AKI. Therefore, the role of biomarkers other than SCr in the early and differential diagnosis of AKI should be explored. For patients with cardiac surgery, some studies have indicated that the novel biomarkers, such as N-acetyl-β-d-glucosaminidase, urinary α1 microglobulin, serum, and urinary neutrophil gelatinase-associated lipocalin (NGAL), glutathione transferase-π, are helpful in identifying AKI. Some urinary biomarkers (interleukin-18 and kidney injury molecule 1) appear to not only predict the accidence of subclinical AKI, which is characterized by an elevation in urinary biomarkers prior to creatinine concentration, but also to have a predictive value in 3-year mortality after cardiac surgery. However, these novel biomarkers to predict AKI are limited for type B aortic dissection patients and need further study in this area.

**Table 1.** AKI as defined by RIFLE, AKIN, and KDIGO.

RIFLE			AKIN			KDIGO		
Stage	SCr	Urine output	Stage	SCr	Urine output	Stage	SCr	Urine output
Risk	SCr increase to 1.5-fold OR GFR decrease > 25% from baseline	<0.5 mL/kg/h for 6 h	1	SCr increase ≥ 26.5 μmol/L (≥ 0.3 mg/dL) OR increase of 1.5-fold to 2.0-fold from baseline	<0.5 mL/kg/h for 6 h	1	Increase in SCr 1.5× to 1.9× baseline OR ≥ 0.3 mg/dL (≥ 26.5 μmol/L) increase	<0.5 mL/kg/h for 6–12 h
Injury	SCr increase to 2.0-fold OR GFR decrease > 50% from baseline	<0.5 mL/kg/h for 12 h	2	SCr increase > 2.0- to 3.0-fold from baseline	<0.5 mL/kg/h for 12 h	2	Increase in SCr 2.0× to 2.9× baseline	<0.5 mL/kg/h ≥ 12 h
Failure	SCr increase to 3.0-fold OR GFR decrease > 75% from baseline OR SCr ≥ 354 μmol/L (≥ 4 mg/dL) with an acute increase of ≥ 44 μmol/L (0.5 mg/dL)	<3 mL/kg/h for 24 h OR Anuria for 12 h	3	SCr increase >3.0-fold from baseline OR SCr ≥ 354 μmol/L (≥ 4.0 mg/dL) with an acute increase of ≥ 44 μmol/L (0.5 mg/dL) OR need for CRRT	<0.3 mL/kg/h for 24 h OR anuria for 12 h	3	Increase in SCr to 3.0× baseline OR increase in SCr to ≥ 4.0 mg/dL OR initiation of CRRT OR in patients < 18 years, decrease in eGFR to < 35 mL/min per 1.73 m	<0.3 mL/kg/h ≥ 24 h OR Anuria ≥ 12 h

CRRT: continuous renal replacement therapy; GFR: glomerular filtration rate; SCr: creatinine

### Pathogenesis

**Figure 1.**
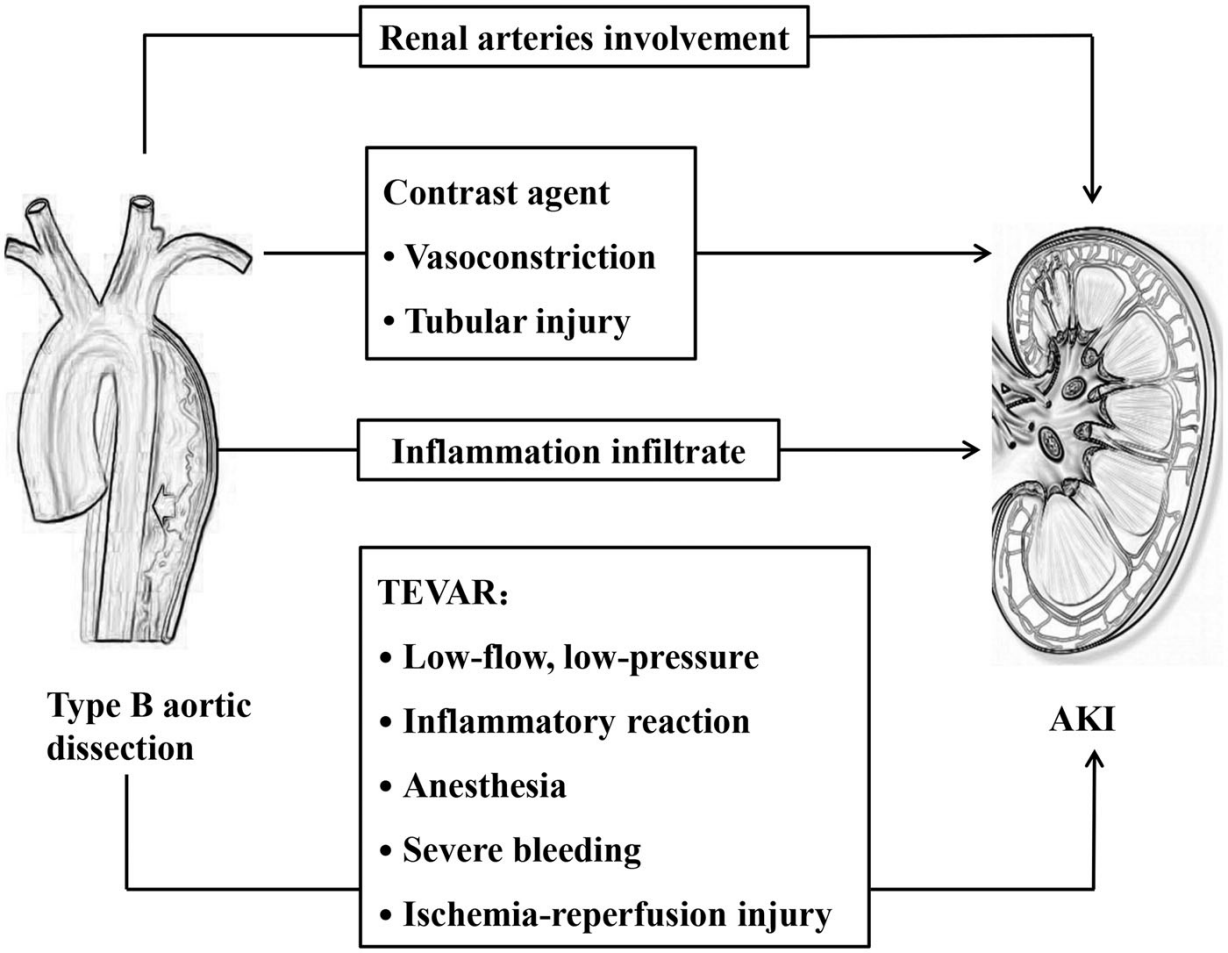
Potential mechanisms of AKI in type B aortic dissection patients.

No specific studies have examined the mechanism of AKI in type B aortic dissection with or without TEVAR, but certain hypotheses exist in the literature. The pathophysiology of AKI might involve multiple factors that intervene in different methods and to differing extents in different patients (Figure 1). Several major injury mechanisms are probably involved in the development of AKI in type B aortic dissection, including renal hypoperfusion, inflammatory response, and contrast agents. All of these injury mechanisms can occur preoperatively, intraoperatively, and postoperatively, or at all of these times and to varying degrees in any given patient.

### Renal hypoperfusion

Renal hypoperfusion is often associated with the presence of type B aortic dissection and TEVAR. Decrease of renal blood flow, whether systemic or localized, played a key role in the pathophysiology of AKI [19,20]. Renal perfusion may be influenced by the blood supply for the side branch of the aorta from the true or false lumen. The obstruction of the dissection flap may result in the occlusions of side branch artery, which can either prolapse from a vessel origin without entering it (dynamic obstruction) or extend directly into a vessel (static obstruction) [21]. Dynamic obstruction may arise due to intermittent occlusion of the branch vessel by protrusion of the intimal flap or false lumen into the ostium of the vessel during the cardiac cycle and represents 80% of malperfusion syndrome patients [22]. Renal arteries were involved in approximately 45% of type B aortic dissection patients [23]. Renal artery involvement, especially if bilateral, causes thrombosis or stenosis, which may directly influence renal perfusion [19]. Renal artery involvement may cause peripheral sympathetic nervous system activity in the kidney, increased blood vessel tension, and reflective increases in the blood pressure and heart rate. Additionally, approximately 80% of aortic dissection patients have a significant burden of hypertension [4], which might further induce expansion of false lumen and increase the fluctuation of the intima, resulting in persistent renal ischemia [19]. Acute renal ischemia can cause severe alterations of the endothelium in small peri-tubule arterioles and capillaries, including postischemic stromal inflammation and microvascular lesions, both of which contribute to sustained renal dysfunction [24]. Additionally, emergency antihypertensive therapy might result in renal impairment, probably due to continued hypoperfusion causing a fall in the glomerular filtration rate. In the case of aneurysmal rupture, emergency TEVAR has the additional burden of hypovolemic shock leading to prerenal renal hypoperfusion and ischemic acute tubular necrosis depending on the duration of hypovolemia. Renal artery blood flow can be improved after TEVAR, however, in some patients, the renal artery is completely supplied by the false lumen with a small incision at the distal end, and increased pressure in the true lumen results in compression of the false lumen after TEVAR, which might lead to a decrease in blood flow to the false lumen or even the possibility of ischemia aggravation. After TEVAR, renal perfusion may improve. However, renal reperfusion injury may then occur and may contribute to AKI by leading to the opening of mitochondrial permeability transition pores in the kidneys, a change that is associated with cell injury or cell death [25]. Moreover, patients with type B aortic dissection are likely to have an obvious burden of intra-aortic mural thrombus, which may be disrupted during device deployment and other endovascular procedures, leading to renal vascular microembolism, and renal parenchyma ischemia [26]. Additionally, anesthesia, severe bleeding, conduction, and rhythm disturbances during TEVAR can trigger hemodynamic instability, and thereby impair renal perfusion.

### Inflammation

Inflammation plays a pivotal role in the development of acute aortic dissection [27,28]. Activated macrophages infiltrate the tunica media and release matrix metalloproteinases (MMPs) and pro-inflammatory cytokines. The excessive production of MMP-1, MMP-9, and MMP-12 leads to the accelerated degradation of collagen and elastin fibers, which causes the remodeling of the aortic wall structure. These inflammatory reactions could impair renal function [1]. Additionally, inflammatory mediators are generated to regulate the inflammatory reaction in impaired kidneys. In turn, the inflammatory response to aortic injury is further enhanced by these inflammatory mediators [19]. Ren et al. indicated that maximum levels of body temperature and white blood cell count were significantly associated with maximum SCr before TEVAR. Additionally, ischemic- and infection-related inflammatory responses are also significant mediators of AKI [9]. Increases in the plasma inflammatory cytokines concentrations are associated with an increased AKI risk and increased in-hospital mortality [16,29]. Komukai et al. reported that maximum C-reactive protein (CRP) was significantly related to impaired oxygenation in acute aortic dissection patients [30]. Sakakura et al. reported that peak CRP level was associated with poor long-term outcomes in type B aortic dissection patients [29]. Moreover, Schillinger et al. reported that an elevated CRP level on admission was a strong predictor of adverse outcome in acute aortic dissection patients [31]. Additionally, for complicated type B aortic dissection patients, tissue injury of TEVAR is associated with the activation of multiple inflammatory pathways and increases pro-inflammatory cytokines levels. Manipulation in the aneurysm during TEVAR may cause white cell activation with the release of various cytokines, such as interleukin-1, interleukin-6, and tumor necrosis factor-α. Additionally, injury to the vascular endothelium may prompt activate protein C, with a subsequent coagulation disorders and loss of cytoprotectivity. Macrophages, neutrophils, and lymphocytes are activated by cytokines and chemokines, and recruited into the renal parenchyma, which caused parenchymal infiltration and thus contributed to AKI.

### Contrast medium

The pathogenesis of contrast-induced AKI is not entirely clear, but it is more likely to involve multiple mechanisms. According to the recent European Society of Cardiology (ESC) guidelines, TEVAR is considered the therapy of choice in complicated type B aortic dissection patients and may be considered along with medical therapy in uncomplicated patients, as well [32]. TEVAR involves the insertion and deployment of a stent graft through the femoral artery. However, the less-invasive nature of TEVAR provides better hospital survival and a less invasive alternative to open surgery for type B aortic dissection. TEVAR has been increasingly used to treat type B aortic dissection. The central principle of TEVAR is to place a covered stent graft over the entry tear on the descending thoracic aorta wall, which drives changes in the hemodynamics of the dissected aorta, leading to depressurization of the false lumen and rapid expansion of the true lumen. However, the contrast agent may be administered more to ensure whether the stent graft is implanted in the true lumen and at the right place. Contrast medium is directly toxic to tubular epithelial cells, resulting in function loss, apoptosis, and necrosis [33]. Indirect mechanisms are concerned with ischemic injury due to vasomotor changes mediated by vasoactive substances, such as nitric oxide, prostaglandins, and endothelin [34]. The relatively low oxygen partial pressure of the outer renal medulla, coupled with increased metabolic demand, makes the medulla particularly vulnerable to the hemodynamic effects of contrast agents [35]. Contrast medium can increase renal tubular viscosity *in vitro*, which results in tubular obstruction and increased interstitial pressure. Increased plasma viscosity raised vascular resistance and thus decreased medullary blood perfusion of the kidney [36]. Particularly in type B aortic dissection patients with preexisting CKD, they have a reduced number of functioning nephrons and an impaired ability to regenerate tubular epithelial cells; thus, using routine average doses of iodinated contrast can result in AKI [37]. Tan et al. [38] indicated that contrast agents had a synergistic effect with hyperglycemia, which could promote the apoptosis of renal cells and lead to the occurrence of AKI. Additionally, inflammatory reactions, oxidative stress, and ischemic injury may be the underlying mechanism of contrast-induced AKI as well [39].

## Risk factors

Most of the information on risk factors of AKI for type B aortic dissection patients comes from retrospective observational studies. These risk factors can be classified as preoperative, intraoperative, and postoperative (Table 2).

**Table 2.** Risk factors of AKI for patients with type B aortic dissection.

Patient-related factors
Chronic kidney disease
Congestive heart failure
Hypertension
Limb malperfusion
Renal malperfusion
Renal artery involvement (bilateral)
Visceral malperfusion
Systolic blood pressure on admission
Electrocardiographic ST-T changes
Serum creatinine on admission (per 0.1 mg/dL)
Blood glucose on admission (per 10 mg/dL)
The relative area change of true lumen
Procedure-related factors
Preoperative factors
Malperfusion complications
Diabetes mellitus
The relative area change of true lumen
Systolic blood pressure on admission > 140 mmHg
Preoperative Hb (1 g/dL increase)
Intraoperative factors
Contrast medium (100 mL increase)
Supra-aortic branches graft bypass hybrid surgery
Postoperative factors
Hemoglobin reduction (1 g/dL increase)

### Preoperative factors

#### Comorbidities

Most of the preoperative AKI risk factors are patient-related, such as preexisting chronic kidney disease (CKD) [9], congestive heart failure [9], hypertension [9,19], and diabetes [40], with CKD being the key player among them. The etiology of CKD is complex and is mainly attributed to chronic hemodynamic insult with low cardiac output and congestion due to history of hypertension and diabetes mellitus. Additionally, chronic conditions adversely affect renal function, significantly increasing the susceptibility to AKI. Zhu et al. reported a positive relationship among CKD, glomerular filtration rate, and AKI for type B aortic dissection patients who received TEVAR. Despite the exclusion of patients with end-stage renal disease, the data showed that over 20% of high-risk patients suffer stage 3 CKD (glomerular filtration rate, 60 mL/min/1.73 m^2^), and approximately 10% of them suffer stage 4 CKD, and the risk of AKI increases significantly at each stage of CKD advancement [10]. Therefore, appropriate caution must be exercised prior to intervention in patients with multiple risk factors.

### Visceral, limb, and renal malperfusion

Malperfusion syndrome is the most frequent complication, with an incidence approaching 30% of cases of aortic dissection [41]. Arterial malperfusion plays a significant role in the development of AKI. Renal malperfusion was the main determinant of AKI development (odds ratio [OR], 3.18; 95% CI: 1.58–6.40, *p* < .001). Additionally, visceral and limb malperfusions are independently predictive of AKI (OR, 2.19; 95% CI: 1.08–4.47, *p* = .031 and OR, 2.18; 95% CI: 1.04–4.61, *p* = .040, respectively), which might attribute this association to the increased ischemic-related inflammatory responses in the perioperative period [9]. Emergent interventions should be employed for type B aortic dissection to prevent death when patients are complicated by malperfusion syndrome.

### Systolic blood pressure (SBP)

Higher SBP on admission is a risk factor of AKI, not only pre-TEVAR [18] but also post-TEVAR [11]. Higher SBP on admission may be associated with renal artery involvement, which activates the renin-angiotensin-aldosterone system and induces a dramatic increase in BP [19]. Additionally, higher SBP acts directly as a parietal stressor and indirectly as a pro-inflammatory trigger, mainly by inducing macrophage recruitment and activation, which causes generalized ischemia of the kidney and the accidence of AKI [11,42]. A report by Luo et al. [11] showed that the cutoff value for SBP on admission >140 mmHg was the most relevant predictive factor of AKI after TEVAR (OR, 2.288; 95% CI, 1.319 − 3.969; *p* = .003). SBP should be carefully monitored in particular, with the aim of lowering the systolic blood pressure to <120 mmHg using intravenous β-blockers (such as labetalol or esmolol) and vasodilators [4]. Additionally, strategies to improve renal perfusion before TEVAR should be considered to prevent AKI exacerbation and to lower the rate of in-hospital complications, such as adequate hydration.

### SCr concentration

Although SCr concentration on admission might have already been influenced by the type B aortic dissection process, it was associated with the incidence of AKI in patients with type B aortic dissection (OR, 1.28; 95% CI, 1.04 − 1.59; *p* = .023) [16]. An increase of SCr concentration may be associated with structural kidney damage or hemodynamic derangements, in turn predicting the high occurrence of AKI after TEVAR. A preexisting change in the kidney function or structure is one of the major susceptibility factors for AKI.

### Change of the relative area of true lumen

Intima dynamic motion before TEVAR might contribute to early recognition of AKI by initial CT angiography (CTA) examination prior to the presence of clinical indicators. Zhao et al. [18] found that patients with relative area of true lumen >42.6% were deemed to have a high incidence of renal ischemia preoperatively, and patients with a large fluctuation of the intima occurred were more common in the AKI group, which had a significantly increased incidence of AKI. These findings are likely to guide early invention for preventing renal function deterioration (such as using minimal effective volumes of contrast medium, non-nephrotoxic drugs, and adequate hydration) in these patients during the perioperative period. Regarding whether early TEVAR could be a preferable renal protective treatment still needs further study. However, this study included a relatively small cohort and might have a relatively large selection bias due to the retrospective design. The results should be confirmed in larger, multicenter, and prospective investigations. Additionally, while intra-aortic hemodynamics and intimal flap motion vary greatly in different regions of the aorta, this study only evaluated endometrial dissection at the superior aspect of the renal artery ostia and its relationship with renal function in this study. More problematic levels should be taken into consideration in future studies.

### Intraoperative factors

#### Contrast medium

Data on the relationship between absolute contrast volume and post-TEVAR AKI are inconsistent. The use of contrast medium before TEVAR does seem to increase the incidence of AKI. Gorla et al. [40] indicated that the use of nephrotoxic contrast media has been associated with renal injury following TEVAR, with the mean contrast medium dose of 356 ± 197 mL. However, the report of Luo et al. did not confirm these results [11], with the mean contrast medium dose of 168.2 ± 46.2 mL, which may explain the discrepancy. Additionally, baseline renal function may need to be taken into consideration, along with contrast volume, to adequately assess insult on viable nephrons. As indicated by Yamamoto et al. [43], patients presenting with impaired kidney function were expected to receive decreased amounts of contrast, which could cause a bias when analyzing its relationship to AKI following TEVAR. Additionally, Gorla et al. [40] also found that patients with malperfusion at admission required more complex procedures, received high loads of contrast medium, were frequently transfused, and therefore developed postoperative AKI more frequently than patients without malperfusion. Given that malperfusion may lead to AKI either through direct effects of renal ischemia or through the release of nephrotoxic metabolites (e.g., myoglobin after reperfusion of a malperfused limb), this result should be interpreted with caution, and the issue should be further investigated in future studies. Nevertheless, more restrictive use of contrast medium if possible in addition to improve perioperative renal protection may help to decrease the incidence of AKI in type B aortic dissection patients. Additionally, in patients with a contrast allergy or renal insufficiency, a magnetic resonance angiogram (MRA) or transesophageal echocardiogram (TEE) can be performed. In a meta-analysis, CT, MRA, and TEE were showed to have similar pooled sensitivity (98–100%) and specificity (95–98%) for the diagnosis of type A and B aortic dissections combined [44]. For diagnosis of type B aortic dissection, a 2002 IRAD study showed sensitivities of 100% for MRA, 93% for CT, 89% for aortography, and 80% for TEE [45]. Despite the lower sensitivity of TEE, it is the second-line imaging modality after CT, as it is faster and easier to obtain in emergency situations than an MRI, which might reduce the incidence of AKI by decreasing the amount of contrast.

### Supra-aortic branch graft bypass hybrid surgery

Hybrid repair of complex aortic arch disease with revascularization of the supra-aortic branches prior to stent graft deployment has evolved as an alternative treatment for selected patients at high risk of conventional open repair [46]. Supra-aortic branch graft bypass surgery before TEVAR for type B aortic dissection patients is performed to prevent posterior circulation ischemia in cases of right vertebral artery dominance with insufficient aortic arch landing zone. Nevertheless, Luo et al. found that type B aortic dissection patients requiring supra-aortic branch graft bypass hybrid surgery had a high risk of AKI (OR, 3.228, 95% CI, 1.526 − 6.831, *p* = .002) [11]. This might be because supra-aortic branch graft bypass hybrid surgery often requires general anesthesia, longer surgical procedure, more blood loss, postoperative transfusion, higher rates of intraoperative hypotension, more severe inflammatory response, and assisted mechanical ventilation, which cause a high incidence of AKI after TEVAR [11].

### Postoperative factors

#### Postoperative hemoglobin (Hb) reduction

Riccardo et al. found that an increased rate of AKI was also observed in patients who had a severe postoperative Hb reduction (>4 g/dL) compared to those with a moderate (2–4 g/dL) or mild Hb reduction (<2 g/dL) for type B aortic dissection patients who underwent TEVAR (OR, 4.34, 95% CI, 1.91–9.85, *p* < .001) [40]. It was reported that anemia after cardiac surgery could induce embolic or ischemic events through inflammatory and free oxygen radicals, leading to acute tubular necrosis and subsequent AKI [47]. Hemodynamic changes should be expected during TEVAR procedures, and renal ischemia induced by a decrease in blood pressure is more likely to occur in patients with anemia than in patients without anemia. Therefore, optimization of baseline factors related to preoperative anemia is important to reduce the development of AKI. Additionally, most influential factors on the Hb reduction may be dissection-related complications (open or contained aortic rupture, gastrointestinal bleeding due to malperfusion syndrome, etc.). Hence, measures aiming at preventing procedure-related bleeding and access site complications as well as a careful monitoring of fluid supply should be taken to avoid hemodilution [40].

## Outcomes

### The requirement of CRRT

CRRT is a direct consequence of AKI associated with high short- and long-term mortality, a decrease in quality of life, and an increase in the costs of treatment. A previous meta-analysis by Coca et al. showed that patients with AKI had a higher risk of developing CKD and end-stage renal dysfunction for patients with cardiac surgery [48]. However, whether it is suitable for type B aortic dissection patients with AKI needs further study. The occurrence of AKI requiring CRRT varies between 6% and 14.6% [9–11], and the discrepancy might be owed to the fact that many studies exclude patients with advanced CKD [9]. For those who develop a new need for hemodialysis, 50% will require dialysis permanently [9].

### Mortality

The occurrence of AKI after TEVAR surgery is related to significantly higher short- and long-term mortality. AKI in type B aortic dissection is independently associated with 30-d mortality among patients experiencing AKI, ranging from 4.7% to 12.5% compared to 0.9% to 2.7% for those without AKI. One-year mortality has ranged from 17.4% to 20% for AKI patients compared to 2% to 10% for patients without AKI. The results show that AKI in type B aortic dissection is associated with 7- to 14-fold increased risk for early (in-hospital or 30-d) mortality and a 3- and 10-fold increased risk for 1-year mortality. Furthermore, the effect of AKI on mortality persists as long as 12 years after hospitalization [9]. Tsai et al. [7] demonstrated that in-hospital renal failure was strongly related to follow-up mortality in patients treated medically, surgically, or with endovascular therapy.

## Management of type B aortic dissection patients with AKI

### AKI prevention

Because of the complex nature of the mechanisms contributing to renal damage for type B aortic dissection patients, prevention strategies applicable to general cardiac disease, general surgery, or percutaneous radiologic interventions could not be completely extrapolated for the prevention of AKI. To date, no trials have studied any specific prevention and treatment strategies in AKI for type B aortic dissection patients. Currently, a reasonable patient-tailored approach focused on limiting risk factors is advised. We have already learned from cardiac surgery experience that the introduction of ‘KDIGO bundle’ can be advantageous [49]. Moreover, the intervention with the supportive care ‘bundle’ should encompass 1) optimization of volume and hemodynamic parameters, which in the case of type B aortic dissection patients means volume optimization before the procedure and blood pressure control; 2) limit of nephrotoxic drugs, such as aminoglycoside antibiotics, nonsteroidal anti-inflammatory agents, angiotensin-converting enzyme inhibitor (ACEI), and angiotensin receptor blockers (ARBs), mainly contrast agents; and, 3) prevention of hypoglycemia. The most important preventive strategy seems to be optimizing renal perfusion before surgery. Using preoperative volume expansion before contrast administration may help minimize contrast-related injury; other modalities, such as the use of bicarbonate or statins could be helpful, but they have not been specifically studied in AKI prevention for type B aortic dissection patients.

Additionally, early implementation of TEVAR may be useful to improve renal malperfusion and thereby reduce the occurrence of AKI. Renal malperfusion caused by type B aortic dissection will yield better results with improvement in the blood supply by the true lumen after TEVAR. Moreover, Penn ABC, a more recent classification for distal dissection that focuses on the evolution of complications in type B aortic dissection, is used to stratify hospital mortality risk and to provide proper management [50,51]. Early TEVAR in the setting of complicated type B aortic dissection patients in Penn class B (the presence of branch-vessel malperfusion), Penn class C (the presence of circulatory collapse), or Penn class B + C yields favorable clinical results. A meta-analysis of outcomes for endovascular treatment of type B aortic dissection reported low rates of in-hospital mortality and major complications of aortic dissections, concluding that endovascular treatment of (complicated) type B aortic dissection is a therapeutic option with favorable initial outcomes. Tien et al. found that for patients with type B aortic dissection in Penn class A (currently termed ‘uncomplicated’, the absence of malperfusion or circulatory collapse), in-hospital mortality was lowest among those treated surgically, at a rate that was much lower than those treated medically, which suggests that early surgical or endovascular repair may confer a mortality benefit to a specific subset of patients with uncomplicated type B aortic dissection [51]. Two-year data from the Investigation of Stent Grafts in Aortic Dissection (INSTEAD) trial showed no difference in all-cause mortality between patients treated with best medical therapy plus stent-graft and those treated with best medical therapy alone [52,53]. However, long-term outcome data support TEVAR for initially uncomplicated type B aortic dissection due to the prevention of late complications and cardiovascular death [54], preemptive TEVAR, if provided at very low risk, is increasingly being used for initially uncomplicated dissection [55,56]. At the 5-year follow-up for INSTEAD-XL, aortic rupture, progression of disease, and vascular mortality were tempered by preemptive TEVAR in the subacute phase of dissection [54]. Thus, in anatomically suitable patients with substantial life expectancy, preemptive TEVAR should be offered irrespective of clinical presentation to prevent late complications. This survival benefit is attributed to the ability of TEVAR to prevent late complications, namely, aneurysmal degeneration, and thereby counterbalance the early hazard associated with intervention. However, more rigorous, randomized data are necessary to confirm these recommendations.

Of note, in type B aortic dissection patients, especially in the area of the abdominal aorta, the risk of progressive aneurysmal degeneration and renal artery stenosis supplied by the false lumen is relatively high after TEVAR [57]. The PETTICOAT (provisional extension to induce complete attachment) technique was to insert a distal bare metal stent into the true lumen of the aorta, distal to the proximal endograft, to stabilize the distal collapsed intimal flap, while allowing blood flow to reno-visceral arteries, which can enhance the effect of the proximal TEVAR in improving the re-expansion of the distal thoraco-abdominal aorta true lumen and thus improve renal perfusion. Additionally, the staged thoraco-abdominal and branch vessel endoluminal repair (STABLE) and stent-assisted balloon-induced intimal disruption and relamination in aortic dissection repair (STABILIZE) techniques, as additions to the PETTICOAT technique, have similar target populations and more aggressive than the PETTICOAT technique. However, due to the lower surgical technical difficulty and safety hazard, the PETTICOAT technique is more widely applied than the STABLE and STABILIZE techniques. The three technologies mentioned above may promote aortic remodeling and improve renal perfusion, thereby reducing the incidence of AKI. However, currently there is insufficient evidence in positive remodeling of the false lumen in the distal aorta and improving short and long-term survival. Therefore, a widespread use of the PETTICOAT technique is unreasonable and it should be limited to patients complicated by dynamic malperfusion [58,59]. Additionally, the relationship between renal artery stenosis and postoperative AKI and the actual effectiveness of stent placement on outcomes such as renal volume loss are to be confirmed further [60].

There are also promising tools on the horizon, which can help in either the prevention or early detection of AKI. Radiologic signs might allow for early detection of visceral malperfusion prior to clinical presentations. Regarding the pathophysiological mechanism of renal malperfusion, the reduction of renal blood flow caused by branch obstruction plays an important role in patients with type B aortic dissection. CTA-based intima dynamic motion measurements can be useful in early AKI detection. Zhao et al. [18] proved that intima dynamic motion may help early detection of renal ischemia by initial CTA examination prior to the presence of clinical indicators. Additionally, a large fluctuation of the intima could increase the incidence of adverse events, especially AKI, postoperatively. The effect of intima dynamic motion on AKI for type B aortic dissection patients remains to be further studied.

### AKI treatment

The treatment of AKI focused on attenuating ischemia, reducing intrarenal inflammation, and supportive care in type B aortic dissection patients. Nutrition is an important component of perioperative care and should not be neglected. Malnourished patients with AKI have an increased risk of mortality [61]. If AKI is severe enough to require CRRT, higher caloric intake by protein supplementation may be necessary [62]. Glycemic control is also suggested in patients developing AKI. Maintaining a glucose concentration ≤150 mg/dL is an appropriate target, while avoiding hypoglycemia (≤80 mg/dL) [63].

CRRT is an effective treatment strategy for AKI patients, especially those who require both circulatory and respiratory support. The current literature on the use of CRRT relates predominantly to patients in the ICU with severe AKI after cardiac surgery. In the absence of data specific to type B aortic dissection patients, clinicians deciding how to approach CRRT in these patients will, inevitably, have to refer to general studies. Currently, the ideal timing of CRRT initiation after cardiac surgery remains uncertain. The KDIGO guidelines suggest that indications for initiating CRRT include the presence of life-threatening changes in fluid levels, electrolytes, and the acid-base balance. A single-center ELAIN trial showed a survival benefit of early CRRT initiation (within 8 h of diagnosis of KDIGO Stage 2) compared with delayed initiation (within 12 h of Stage 3 AKI or no initiation) among critically ill patients with AKI [64]. However, owing to the limited number of studies addressing the timing of CRRT initiation, the impact of early initiation of CRRT on type B aortic dissection patients with AKI outcome remains uncertain and needs further study.

## Conclusion

AKI is a one of the common complications of type B aortic dissection. The occurrence of AKI increased patient short- and long-term mortality. Early identification of high-risk patients, early diagnosis of AKI, stabilization of the hemodynamic parameters, avoidance of nephrotoxic drugs, and optimization of the use of contrast agents are the major strategies for the reduction of AKI in type B aortic dissection patients. However, most current studies are limited by their small sample size, their retrospective nature, and the heterogeneity of the analysis results. Therefore, further large clinical trials assessing the potential risk factors of AKI and effective methods for the prevention and treatment of AKI remain priorities for type B aortic dissection patients.

## Data Availability

The data that support the findings of this study are available on request from the corresponding author.
